# Host-shift as the cause of emerging infectious diseases: Experimental approaches using Drosophila-virus interactions

**DOI:** 10.1590/1678-4685-GMB-2020-0197

**Published:** 2020-11-23

**Authors:** André C. Pimentel, Camila S. Beraldo, Rodrigo Cogni

**Affiliations:** 1 Universidade de São Paulo, Instituto de Biociências, Departamento de Ecologia, São Paulo, SP, Brazil.; 2 University of Helsinki, Organismal and Evolutionary Biology Research Program, Helsinki, Finland.

**Keywords:** Wolbachia, evolution, infection, cooccurrence, virus diversity

## Abstract

Host shifts, when a cross-species transmission of a pathogen can lead to successful infections, are the main cause of emerging infectious diseases, such as COVID-19. A complex challenge faced by the scientific community is to address the factors that determine whether the cross-species transmissions will result in spillover or sustained onwards infections. Here we review recent literature and present a perspective on current approaches we are using to understand the mechanisms underlying host shifts. We highlight the usefulness of the interactions between *Drosophila* species and viruses as an ideal study model. Additionally, we discuss how cross-infection experiments — when pathogens from a natural reservoir are intentionally injected in novel host species— can test the effect cross-species transmissions may have on the fitness of virus and host, and how the host phylogeny may influence this response. We also discuss experiments evaluating how cooccurrence with other viruses or the presence of the endosymbiont bacteria *Wolbachia* may affect the performance of new viruses in a novel host. Finally, we discuss the need of surveys of virus diversity in natural populations using next-generation sequencing technologies. In the long term, these approaches can contribute to a better understanding of the basic biology of host shifts.

## Introduction

In less than eight months, COVID-19 has spread from a few cases in Wuhan, China, to more than eighteen million people in almost everywhere in the world (Coronavirus Research Center, https://coronavirus.jhu.edu/map.html  visited on August 6th, 2020). The disease is caused by a new human-infecting virus, SARS-CoV-2 (Huang *et al.*, 2020; Sironi *et al.*, 2020). Phylogenetic analyses suggest that the natural host of this virus is likely bats, and that a possible wild animal sold at the Wuhan food market might be an intermediate host that helped transmission to humans (Lu *et al.*, 2020). This is a classic example of an emerging infectious diseases (EID) — infections recognized in a host population for the first time (Morens and Fauci, 2013). The causes of the emergence of novel diseases are pointed out as due to multiple factors, which may involve socio-economic, environmental, and ecological components (Jones *et al.*, 2008). 

As in the case of COVID-19, a common cause of EID is the cross-species transmission of pathogens, which can lead to sustained onwards transmission. This successful pathogen emergence may occur through two different processes that vary in the level of pathogen adaptation following the colonization: host range expansion and host shift. Expansion of host range occurs when the jump increases the number of host species that the pathogen is able to infect without changing pathogen’s original gene pool (Thines, 2019). In turn, host shift takes place when the jump increases genetic differentiation in the pathogen, leading to specialization on the novel host (Longdon *et al.*, 2014; Choi and Thines, 2015; Thines, 2019). In the case of SARS-CoV-2, the recognition of the virus receptor (angiotensin-converting enzyme 2, ACE2) is a feature shared with its relative viruses (e.g. SARS-CoV and the bat virus SL-CoV WIV16) that allowed the jump to humans (Yang *et al.*, 2016; Lu *et al.*, 2020). Furthermore, an amino acid residue substitutions in SARS-CoV-2 spike protein increases binding affinity to human ACE2 (Wang *et al.*, 2020), what may indicate specialization on human host. Pathogen host shifts are often observed in humans, in which 60.3% of EID are zoonoses, changing mainly from wild animal reservoirs (Jones *et al.*, 2008). Some examples include the acquired immunodeficiency syndrome (AIDS) pandemic caused by the human immunodeficiency virus (HIV), which jumped into humans from non-human primates (Sharp and Hahn, 2011; Faria *et al.*, 2014), and Ebola, whose virus shifted from fruit bats to humans (Leroy *et al.*, 2005).

One of the biggest current challenges to epidemiologists is to address the factors that guarantee the success of cross-species transmissions, leading to host shifts. By addressing the mechanisms of host shift, it would be possible to understand what causes spillover infections (i.e. events with no or short onward transmission) and what leads to sustained infections, when the pathogen enters, replicates itself within and is transmitted between members of the new host species (Longdon *et al.*, 2014; Engelstädter and Fortuna, 2019).This would allow scientists to anticipate potential epidemics places, reducing the economic, environmental and social burden. Predicting the spatiotemporal occurrence of a host shift is still challenging, as it may be linked to a multitude of variables ranging from host and pathogen geographic dispersion to changes in host phenotype and genetics (Woolhouse *et al.*, 2005). Assessing the factors favoring host shifts and identifying potential susceptible taxa is crucial to novel emerging pathogen research as well as to mitigate their impacts (Woolhouse *et al.*, 2005; Burbrink *et al.*, 2017).

Here we discuss how experimental approaches can help our comprehension of mechanisms favoring host shifts. We highlight the usefulness of the interactions between *Drosophila* species and viruses as a study model and review recent advances and current methods being pursued. We claim that understanding the basic biology of host shifts is essential to prevent and deal with infectious diseases such as COVID-19.

## The advantages of the *Drosophila*-virus model

Studied for more than a century, *Drosophila melanogaster* has become the most studied organism in many fields of biology. Most of its success as a model organism is due to its rapid generation time, small size, easy stock maintenance, and unrivaled availability of genetic and genomic tools (Powell, 1997). Another advantage of this model in many fields, including studies of host-virus interactions, is its high degree of evolutionary conservation with other animals (Hoffmann, 2003; Lemaitre and Hoffmann, 2007; Panayidou *et al.*, 2014; Xu and Cherry, 2014). Many defense mechanisms against viruses in *Drosophila* are conserved in vertebrates, such as Toll, Imd, and Jak-Stat pathways (Lemaitre *et al.*, 1996; Merkling and Van Rij, 2015; Marques and Imler, 2016). However, a disadvantage of this system is that some immune pathways are restricted to some taxa and are not directly comparable to *Drosophila*. For example, *Drosophila* lacks an adaptive immune system, an important response to several pathogens that ensures immunological memory in vertebrates (Flajnik and Kasahara, 2010).

There are well established protocols for experimental work on *Drosophila*-virus interactions (Merkling and Van Rij, 2015; Yang *et al.*, 2019) and recent research has covered diverse aspects of host-virus biology. Studies have looked at the genetic architecture of resistance to virus infection, including the identification of many major effect genes that affect resistance and their mechanisms of antiviral action (Magwire *et al.*, 2012; Cogni *et al.*, 2016; Cao *et al.*, 2017). Some works have performed experimental tests of host-shifts in a controlled phylogenetic design (Longdon *et al.*, 2011, 2015). *Drosophila* has also been used as a model to understand the replication mechanisms of human viruses such as SARS-CoV (Hughes *et al.*, 2012). Additionally, research on the diversity of insect viruses, and the mechanisms that control virus infections, have the potential to discover new adaptations that can inspire the development of novel antiviral strategies by the pharmaceutical industry (Olmo *et al.*, 2019). 

## Experiments on host-shift

**Figure 1 -:**
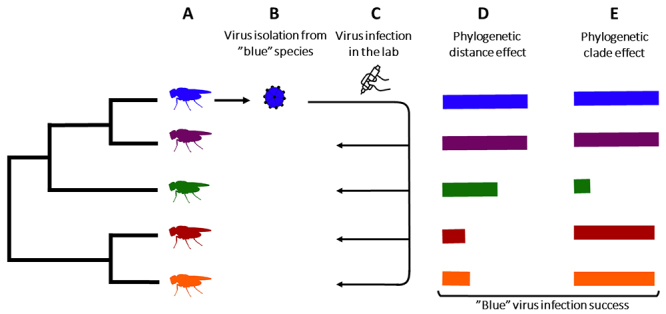
Cross-infection experiment and possible ways in which host phylogeny affects virus’ shift. (A) Hypothetical host phylogeny. The flies’ shading in various colors at the tips of the tree indicate different host species. (B) Isolation of a natural virus occurring in one of the host species (blue). (C) Artificial infection of the virus isolated in other host species (purple, green, red, and yellow). (D/E) Bars represent the virus infection success in each host species, and colors indicate the host species corresponding to each bar. (D) The virus’ infection success decreases as the host relatedness to its natural host natural host species increases (phylogenetic distance effect). (E) The virus’ infection success is not related to the phylogenetic proximity to its natural host species, but there are susceptible and resistant clades scattered across the host phylogeny (phylogenetic clade effect).

Cross-infection experiments — when pathogens from a natural reservoir are intentionally injected into new host species — are used to simulate host shifts and have been described as fruitful practices to understand mechanisms underlying host-pathogen interactions (Figure 1). Although the enormous theoretical efforts done to uncover which factors lead to sustained or to short chain infections (Chabas *et al.*, 2018; Bonneaud *et al.*, 2019; Dallas *et al.*, 2019; Engelstädter and Fortuna, 2019), we lack system-related information not observable in nature, such as the frequency of cross-species pathogen transmissions, and the likelihood of infection given the exposure of the host (Mollentze *et al.*, 2020). Data of this nature are only obtained through experimental studies. In order to better evaluate the array of possible data resulting from cross-infection experiments, it is important to categorize the components of the interaction. Infection dynamics depends on host effects (e.g. susceptibility and defense mechanisms), pathogen effects (e.g. replication ability and virulence), and interaction effects, which is related to the synergy between host and pathogen inherent features (Mollentze *et al.*, 2020). 

Host susceptibility can be dismantled in a set of attributes specific to the species and/or the individual, such as genetics, immunity, microbiome, age and sex (Casadevall and Pirofski, 2017). Conversely, during an infection, hosts may use a combination of two different mechanisms to defend against pathogens, resistance and tolerance (Ayres and Schneider, 2012). Resistance is when there is an activation of host's immune system to control pathogen's replication, and tolerance, when the host is able to avoid a decrease in its own fitness without necessarily altering the parasite load (Schneider and Ayres, 2008; Ayres and Schneider, 2012; Medzhitov *et al.*, 2012; Vale *et al.*, 2016). 

The susceptibility of potential hosts varies greatly within and between taxa, and a key factor predicting it is the phylogenetic relatedness among potential hosts (De Vienne *et al.*, 2013; Longdon *et al.*, 2014; Engelstädter and Fortuna, 2019). This phylogenetic influence may occur through phylogenetic distance effect or phylogenetic clade effect (Figure 1; Longdon *et al.*, 2014, 2015). Phylogenetic distance effects suggest that a pathogen infection success decreases as the phylogenetic distance from the natural host increases (Longdon *et al.*, 2014). In such case, taxa phylogenetically closer to the natural host are more likely to be infected (Corey and Waite, 2007; De Vienne *et al.*, 2009). For instance, Longdon *et al.* (2011) examined the variation in persistence and replication of three sigma viruses, isolated from different species of *Drosophila*, in 51 Drosophilidae novel hosts. They demonstrated that the viruses’ replication ability was negatively related to the phylogenetic distance from the donor host. Considering that in novel infections the virus requires adaptations to use the cellular machinery of the new host, and supposing such structural changes increase with evolutionary divergence time, shifts to phylogenetically more distant hosts will demand more adaptations. Hence, being less able to replicate themselves, those three sigma viruses presented lower viral titers in species phylogenetically distant from their natural host (Longdon *et al.*, 2011). 

Phylogenetic clade effects predict that pathogen infection success varies between different host clades, but is similar within them — i.e. a particular clade of hosts may have related susceptibility to the pathogen, independent of its phylogenetic distance from the natural host (Longdon *et al.*, 2014, 2015). This occurs when particular clades share some features that made them particularly resistant or susceptible to the pathogen (Longdon *et al.*, 2014). For example, in a cross infection experiment using Drosophila C virus (DCV) and 48 species of *Drosophila*, Longdon *et al.* (2015) did not observe distance effect on viral load, but a pattern in which titers were clustering together across the host phylogeny. They hypothesized that physiological, immunity or molecular host features driving the virus infection success could be distributed heterogeneously among clades, generating “patches” of hosts with high susceptibility throughout the phylogeny.

Regarding the pathogen, virulence is a crucial trait to consider in host shift studies. Virulence is the cost in fitness a pathogen causes to its host due to infection (Read, 1994; Vale *et al.*, 2016, 2018), and it may vary following a host shift, presenting high levels in particular species and leading to outbreaks and epidemics (Woolhouse *et al.*, 2005; Jones *et al.*, 2008). Initially, virulence was thought to be a direct consequence of parasite replication, being linked to the idea that the host-parasite interaction evolves towards avirulence, i.e. the pathogen does not cause a cost in fitness for the host anymore (Alizon *et al.*, 2009). However, host susceptibility features, e.g. resistance or tolerance, may affect how virulent a pathogen could be, decoupling virulence and pathogen load measures (Gandon and Michalakis, 2000; Gandon, 2002). 

Recent studies have looked at the interaction between host susceptibility and pathogen virulence. An elucidative example is the meta-analysis of cross-species tests that was developed by Mollentze *et al.* (2020). They analyzed the progression of rabies virus inoculations from bats and carnivores in other mammal species. This research showed that virus incubation period was longer in receptive hosts with higher body temperature. Interestingly, host body temperature for those groups analyzed were not correlated with phylogenetic distance, but tended to cluster across the phylogeny. They argue that mismatches between hosts physiological features and their evolutionary history may be influencing the infection progression and the success of cross-species transmissions. 

As each host-pathogen association has its specificities and particular interaction results, it is imperative to compare, in a systematic manner, the effects of different infections on the fitness of both host and pathogen. This approach contributes to unravel factors driving the variation of host susceptibility, and pathogen’s virulence and replication capacity. We are using this approach by isolating common viruses in field populations that vary in virulence, and manually injecting them into new hosts (as in Longdon *et al.*, 2011, 2015). In the mid- and long-terms (hyphen and plural), such empirical data are useful to generate parameter distributions to model factors favoring host shifts, and to identify general rules promoting the emergence of infection diseases.

## Virus co-occurrence

**Figure 2 -:**
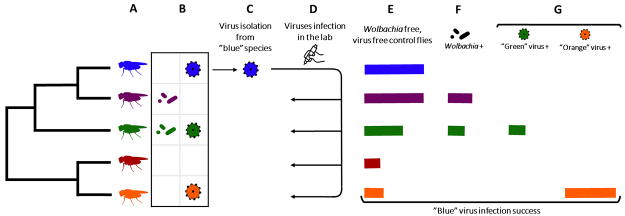
Possible effects of *Wolbachia* and natural virus community on new hosts species on virus’ shift. (A) Hypothetical host phylogeny. The flies’ shading in various colors at the tips of the tree indicate different host species. (B) Two host species (purple and red) naturally carry the bacteria Wolbachia (first column). Three species (blue, green and orange) naturally carry three different viruses - blue, green and orange (second column). (C) Isolation of a natural virus occurring in one of the host species (blue). (D) Artificial infection of the virus isolated in other host species (purple, green, red, and yellow). (E-G) Bars represent the virus infection success in each host species, and colors indicate the host species corresponding to each bar. (E) Virus’ infection success in laboratory control lines free of *Wolbachia* and other viruses. (F) When *Wolbachia* is present in the host, the blue virus’ infection success is lower. (G) When the green virus is present in a host, the blue virus’ infection success is lower, but when the orange virus is present in the host, the blue virus’ infection success is higher.

Viruses do not occur in isolation inside their hosts. After the cross-species transmission, the virus needs to interact with the natural viral community already present in the novel host. Prevalence of viruses in insects is far from negligible, and can reach more than 80% for a given virus, depending on the sampled locality (Webster *et al.*, 2015). In a host-shift context, high prevalence of an endemic virus in the new host can directly affect the fitness of the virus that switched hosts (Figure 2). The co-occurrence of viruses may result in three different outcomes. First, there may be inhibition of viral replication if there is competition for host resources (viral interference) (Salas-Benito and De Nova-Ocampo, 2015). Second, if the presence of one virus compromises the host immune systems, the replication of the other virus may be favored (Kuwata *et al.*, 2015). Third, there may be an apparent absence of fitness consequences (viral accommodation) (Salas-Benito and De Nova-Ocampo, 2015). Considering the specificities of the natural viral community inside potential hosts, it is essential to understand how the dynamics between different viruses affects the occurrence of host shifts.

The presence of a given virus can negatively affect a second infection if both depend on the same host resources, i.e. endemic viruses restrict cellular resources availability for the novel infecting virus. For instance, cell experiments with dual infections had shown that insect-specific viruses can inhibit the growth of Zika, dengue and La Crosse virus (Schultz *et al.*, 2018). In *Aedes aegypti* mosquitos co-infected with two dengue viruses strains - DENV-1 and DENV-4 -, there was a competitive displacement of DENV1 by DENV-4, and only DENV-4 was detected in mosquito salivary gland, improving its transmission potential in a cooccurring event (Vazeille *et al.*, 2016). This can have consequences on virus strains displacement in dengue epidemics (L'Azou *et al.*, 2014). Therefore, this coinfection approach also provides insights for the arbovirus’s transmission and prevalence, which currently impacts human health.

Regarding human respiratory viruses, there are epidemiological data supporting viral interference (e.g. Linde *et al.*, 2009). A well-documented example is the interference between influenza viruses. Infection with influenza virus A(H1N1)pdm09 prevents subsequent infection with a different influenza type, causing temporary immunity following the first infection (Kelly *et al.*, 2010; Laurie *et al.*, 2015). Even though COVID data are preliminary, an equivalent viral interference may occurs, since SARS-CoV-2 patients are infrequently co-infected with other respiratory viruses (Blasco *et al.*, 2020; Nowak *et al.*, 2020).

An alternative scenario to competition is when a virus can benefit from natural infections. For example, when *Culex tritaeniorhynchus* cell line is previously infected by Culex flavivirus, subsequent infection with dengue virus enhances dengue viral titer in late stages of infection (Kuwata *et al.*, 2015). This outcome is probably due to Culex flavivirus action on host antiviral defense. By expressing viral suppressor of RNAi, the virus decreases immune response and favors new infections or higher replication rates (Berry *et al.*, 2009; Palmer *et al.*, 2018). A third scenario is when virus fitness is not affected by cooccurring viruses. For instance, co-infection of *A. aegypti* cells with Zika and chikungunya viruses did not affect replication of the two viruses (Goertz *et al.*, 2017). This lack of interference between both viruses may be explained by the different subcellular fractions occupied by these viruses during their replication (Goertz *et al.*, 2017). Overall, interactions between viruses can lead to different outcomes in fitness of the new infecting virus, affecting the chances of a host-shift.

Surprisingly, these possible interaction effects of cooccurring viruses have not been tested in *Drosophila melanogaster* (Palmer *et al.*, 2018). We propose an experimental approach with laboratory-controlled superinfections, in which individual flies are previously infected with a sub lethal dose of a virus, and afterwards infected with a second virus. The replication rate and virulence of the second virus indicate what would be expected in a host-shift in which the host has a high natural prevalence of a virus.

## *Wolbachia* virus blocking

Not only can virus co-occurrence affect the result of a host-shift, but interactions with other organisms can also do so (Figure 2). A classic example is the presence of the bacterial endosymbiont *Wolbachia* which plays a multitude of effects on host fitness, such as protection against viral infection. *Wolbachia* is an alphaproteobacterium that lives within the cytoplasm of arthropod cells, and is maternally transmitted to the offspring. Until the recent past, it was viewed primarily as a parasite that manipulates host reproduction, most commonly by inducing cytoplasmic incompatibility (Bourtzis *et al.*, 1996). Cytoplasmic incompatibility allows *Wolbachia* to invade populations by causing embryonic mortality when uninfected females mate with infected males, thus conferring a selective advantage to infected females (Turelli and Hoffmann, 1991; Werren *et al.*, 2008).

More recently, basic research on *Drosophila-*virus interactions has discovered that *Wolbachia* can protect *Drosophila* species against infection by RNA viruses (Hedges *et al.*, 2008; Teixeira *et al.*, 2008). The applied potential of this finding to control arboviruses was soon noticed in the scientific community. Combined with *Wolbachia*’s ability to invade populations due to cytoplasmic incompatibility, this provides a way to modify natural mosquito populations, turning them resistant to viral infections. *Wolbachia* has been transferred from *Drosophila* to the mosquito *Aedes aegypti*, where it limits the replication of arboviruses (Moreira *et al.*, 2009). When *Wolbachia* infected mosquitoes were released into the wild, the bacterium spread through the mosquito populations by cytoplasmic incompatibility (Hoffmann *et al.*, 2011; Walker *et al.*, 2011). Large field trials have shown that this approach can decrease dengue prevalence in human populations (Indriani *et al.*, 2020; Ryan *et al.*, 2020). A great advantage of this method to control arboviruses is that *Wolbachia* can block the replication of not only dengue virus, but also chikungunya, yellow fever, Zika and West Nile viruses (Moreira *et al.*, 2009; Glaser and Meola, 2010; van den Hurk *et al.*, 2012; Aliota *et al.*, 2016). 

The long-term success of this strategy depends on the knowledge of basic ecological and evolutionary aspects of virus blocking by *Wolbachia*. For example, there is great variation among *Wolbachia* lineages isolated from different *Drosophila* species in their ability to control virus infection (Martinez *et al.*, 2014). We are expanding this study by investigating virus protection ability on a diverse set of *Wolbachia* lineages and testing if protection works with different viruses. Another important aspect that has not been completely understood yet is if *Wolbachia* protects against virus infection in wild populations of *Drosophila*. There is some evidence that this may not be the case. It seems that there was no association between virus incidence and *Wolbachia* presence in natural *D. melanogaster* populations (Webster *et al.*, 2016; Shi *et al.*, 2018), but these studies may have low statistical power due to limited sample size. We plan to investigate this further to test if virus blocking in the natural *Drosophila* host occurs in wild populations or if it is only a laboratory phenomenon. If virus protection occurs in natural populations, it may have important ecological and evolutionary implications, such as changing the selective pressure on host resistance genes (Martinez *et al.*, 2016; Faria *et al.*, 2018). Finally, phylogenetic experiments on host-shift as described above, can be repeated with species that naturally host *Wolbachia,* to test how the presence of this endosymbiont may affect replication of the new virus on different host species (Figure 2). 

## Virus diversity in natural populations

**Figure 3 -:**
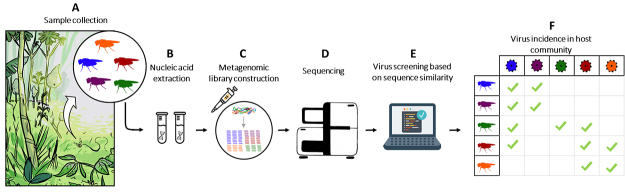
Virus diversity in natural populations. (A) Fly natural populations are collected in the field and sorted into different species. (B) Nucleic acid extraction in the laboratory. (C) Metagenomic library construction. (D) Use of next generation sequencing of the libraries. (E) Bioinformatic work on virus screening based on sequence similarity. (F) Matrix showing incidence of each virus on each host species.

Another essential piece of information to understand host shifts is the knowledge of virus natural host range and frequency of cross-species transmissions in wild populations. This can be obtained by comprehensive surveys of virus diversity in related hosts (Figure 3). Historically, virus diversity was only studied on disease-causing viruses in human and economically important species. Recently, however, the use of metagenomics in diverse taxonomic groups has revolutionized our view of the RNA virosphere as much more phylogenetically and genomically diverse than previously though (Shi *et al.*, 2016; Obbard, 2018; Zhang *et al.*, 2018, 2019). These new studies have uncovered the relative importance of virus-host codivergence versus host-shifts, and showed that host-shifts are common and most of the times not associated with diseases in the new host (Zhang *et al.*, 2019).

In *Drosophila,* a seminal paper in 2015 (Webster *et al.*, 2015) used metagenomics in wild *D. melanogaster* populations and identified more than 20 new viruses. They also used the presence of virus-derived 21 nucleotide (nt) small RNAs, a characteristic response of the RNAi antiviral defenses in *Drosophila* (Wang *et al.*, 2006), to confirm that the virus sequences found were active virus infections. They found that viruses are common in wild populations, and also in laboratory stock lines and cell culture (by using publicly available RNA datasets). Webster et al. (2016) used a similar approach in six *Drosophila* species common in the UK and found 25 novel viruses. Interestingly, they found that few viruses are generalists, being able to infect different host species, and that many viruses shared among closely related species within the *D. obscura* group were less likely shared among more distantly related hosts. These results indicate a high diversity and incidence of viruses in natural populations and that most viruses are host specialists.

We are studying virus diversity in native drosophilid communities collected in the Atlantic Forest of Brazil. By using metagenomics in wild collected flies, we plan to discover new viruses and compare the virus diversity with the few previously studied *Drosophila* species (Webster *et al.*, 2015, 2016). The Atlantic Forest drosophilid communities are highly diverse and contain species from different radiations forming a mix of common species that are close or distant related phylogenetically (Döge *et al.*, 2008). This is the ideal situation to investigate host range and level of specialization of the different viruses and to contrast scenarios of codivergence or host shifts. This survey will likely give interesting virus candidates to be isolated and subsequently used in the experimental approaches described above.

## Conclusion

Host shifts are complex phenomena affected by a multitude of factors and are the main cause of emerging infectious diseases such as COVID-19. Therefore, making predictions about the emergence of novel infections is extremely hard once factors driving this process are not entirely understood. In addition, we lack specific data essential for such forecast. We are applying diverse approaches using the interaction between *Drosophila* species and viruses, including cross-infection experiments in a phylogenetic controlled context, experiments testing the effects of virus cooccurrence and virus blocking by the bacteria *Wolbachia*, and surveys of virus diversity in natural populations using next-generation sequencing technologies. We argue that these practices provide a better understanding of the basic biology of host shifts, contributing to the identification of general rules favoring the emergence of infectious diseases in the long term.
